# Genetic variants in ultraconserved regions associate with prostate cancer recurrence and survival

**DOI:** 10.1038/srep22124

**Published:** 2016-02-23

**Authors:** Bo-Ying Bao, Victor C. Lin, Chia-Cheng Yu, Hsin-Ling Yin, Ta-Yuan Chang, Te-Ling Lu, Hong-Zin Lee, Jiunn-Bey Pao, Chao-Yuan Huang, Shu-Pin Huang

**Affiliations:** 1 Department of Pharmacy, China Medical University, Taichung, Taiwan; 2 Sex Hormone Research Center, China Medical University Hospital, Taichung, Taiwan; 3 Department of Nursing, Asia University, Taichung, Taiwan; 4 Department of Urology, E-Da Hospital, Kaohsiung, Taiwan; 5 School of Medicine for International Students, I-Shou University, Kaohsiung, Taiwan; 6 Division of Urology, Department of Surgery, Kaohsiung Veterans General Hospital, Kaohsiung, Taiwan; 7 Department of Urology, School of Medicine, National Yang-Ming University, Taipei, Taiwan; 8 Department of Pharmacy, Tajen University, Pingtung, Taiwan; 9 Department of Pathology, Kaohsiung Medical University Hospital, Kaohsiung, Taiwan; 10 Department of Pathology, Faculty of Medicine, College of Medicine, Kaohsiung Medical University, Kaohsiung, Taiwan; 11 Department of Occupational Safety and Health, China Medical University, Taichung, Taiwan; 12 Department of Pharmacy, Linsen Chinese Medicine Branch, Taipei City Hospital, Taipei, Taiwan; 13 Department of Urology, National Taiwan University Hospital, College of Medicine, National Taiwan University, Taipei, Taiwan; 14 Department of Urology, Kaohsiung Medical University Hospital, Kaohsiung, Taiwan; 15 Department of Urology, Faculty of Medicine, College of Medicine, Kaohsiung Medical University, Kaohsiung, Taiwan; 16 Graduate Institute of Medicine, College of Medicine, Kaohsiung Medical University, Kaohsiung, Taiwan

## Abstract

Ultraconserved regions (UCRs) are DNA segments of longer than 200 bp in length that are completely conserved between human, rat, and mouse genomes. Recent studies have shown that UCRs are frequently located at fragile sites involved in cancers, and their levels of transcription can be altered during human tumorigenesis. We systematically evaluated 14 common single-nucleotide polymorphisms (SNPs) within UCRs in three cohorts of prostate cancer patients, to test the hypothesis that these UCR SNPs might influence clinical outcomes. Examination using multivariate analysis adjusted for known clinicopathologic factors found association between rs8004379 and recurrence in localized disease [hazard ratio (HR) 0.61, 95% confidence interval (CI) 0.41–0.91, *P* = 0.015], which was confirmed in the replication set (HR 0.70, 95% CI 0.51–0.96, *P* = 0.027). Remarkably, a consistent association of rs8004379 with a decreased risk for prostate cancer-specific mortality was also observed in the advanced prostate cancer patient group (HR 0.48, 95% CI 0.32–0.70, *P* < 0.001). Additional *in silico* analysis suggests that rs8004379 tends to affect *NPAS3* expression, which in turn was found to be correlated with patient prognosis. In conclusion, our findings suggest that SNPs within UCRs may be valuable prognostic biomarkers for assessing prostate cancer treatment response and survival.

Ultraconserved regions (UCRs) are extreme representatives of conserved non-coding genomic sequences, which are longer than 200 bp and are fully conserved between orthologous regions of the human, rat, and mouse genomes^1^. Selection maintaining these sequences over millions of years of evolution suggests that these regions might contain functional elements that are required for cell survival^2^. Examining the genomic localization of UCRs has shown that some overlap with coding exons, but the majority are located in introns or intergenic regions. Studies have shown that exonic UCRs are enriched in genes involved in RNA binding and splicing regulation, and might be critical for maintaining splicing factor expression. The non-exonic UCRs tend to cluster near transcription factors and developmental genes, where they have been suggested to be involved in the transcriptional regulation by acting as enhancers for distal neighboring genes^1^. UCRs are often situated at fragile sites and cancer-associated genomic regions. Most importantly, a high percentage of UCRs can be transcribed generating a class of long non-coding RNAs, exhibiting expression patterns that are frequently altered in human carcinomas, including prostate cancer^3^,^4^.

Prostate cancer is one of the most commonly diagnosed cancers in men. Although the incidence of prostate cancer is lower in Asians than in other ethnic groups, its incidence has risen rapidly and it has become one of the major cancer-related deaths in Asia^5^. Radical prostatectomy (RP) and androgen-deprivation therapy (ADT) are the most common treatment options for localized and advanced prostate cancer, respectively. Despite the initial efficacy of treatment, a subset of patients eventually develops recurrent disease. Thus, there is an urgent need to identify novel biomarkers that can guide patient management.

Given UCRs have been subjected to selection resulting in their absolute conservation between human, mice, and rats, we hypothesized that single-nucleotide polymorphisms (SNPs) within UCRs might harbor important biological functions that might modulate cancer progression. In this study, we identified 14 common SNPs (minor allele frequency, MAF > 0.05) from analysis of 481 known UCRs^1^, and then conducted a multi-stage study to investigate whether there were any associations between these UCR SNPs and prostate cancer progression and mortality.

## Results

### Characteristics of the participants

The basic prostate cancer patient characteristics are shown in Table 1. For the localized prostate cancer cohort, 75 (30.5%) patients experienced biochemical recurrence (BCR) during the median follow-up periods of 50 months in the discovery dataset, and 109 (51.4%) experiencing BCR over 60 months of follow-up in the replication dataset. Overall factors with an effect on BCR rate include prostate-specific antigen (PSA), pathologic Gleason score, and stage (*P* < 0.001). In the advanced prostate cancer cohort, 94 (18.7%) patients died from prostate cancer over a median follow-up period of 60 months. PSA level at ADT initiation, Gleason score, stage, PSA nadir, time to PSA nadir, and treatment modality were all associated with prostate cancer-specific mortality (PCSM) (*P* ≤ 0.002).

### Individual SNPs and clinical outcomes

**Figure 1:**
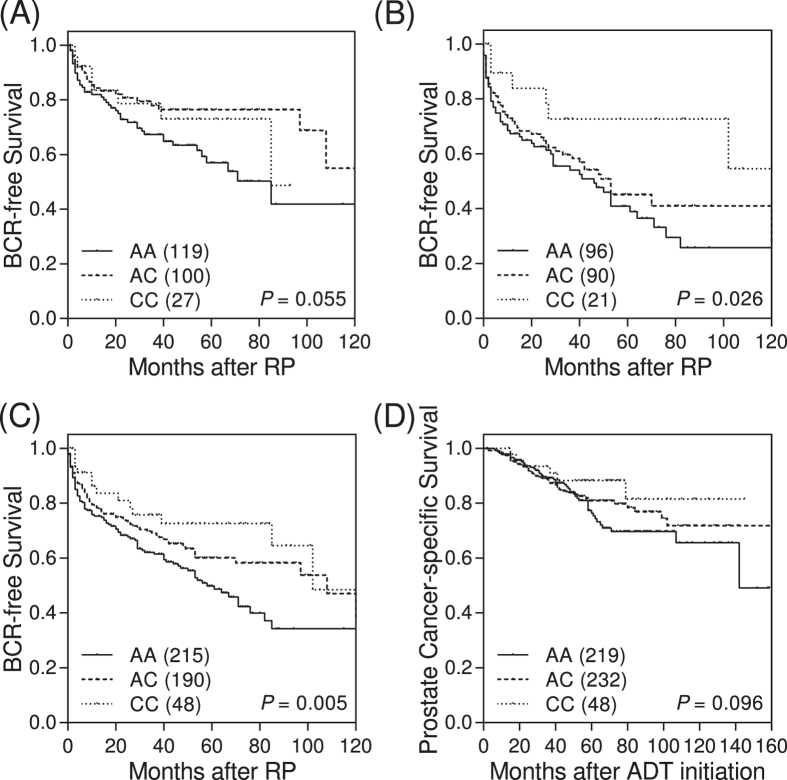
Impact of uc.368 rs8004379 on prostate cancer progression. Kaplan-Meier estimates of BCR-free survival for uc.368 rs8004379 genotypes in localized prostate cancer patients receiving RP from the (**A**) discovery cohort, (**B**) replication cohort, and (**C**) combined analysis. (**D**) Kaplan-Meier estimates of prostate cancer-specific survival for advanced prostate cancer patients receiving ADT by uc.368 rs8004379 genotypes. Numbers in parentheses indicate the number of patients.

Of the 14 UCR SNPs we analyzed, only the SNP rs8004379 in the UCR uc.368 showed a significant association with BCR when examining the discovery dataset of localized prostate cancer patients (*P* = 0.020, Supplementary Table S1). The variant allele, C, for rs8004379 was associated with a decreased risk of BCR in a dose-dependent manner after adjusting for age, PSA level, pathologic Gleason score, and stage [per-allele hazard ratio (HR) 0.61, 95% confidence interval (CI) 0.41–0.91, *P* = 0.015; Table 2 and Fig. 1]. This association was confirmed in the replication dataset (HR 0.70, 95% CI 0.51–0.96, *P* = 0.027), and in the combined analysis (HR 0.66, 95% CI 0.52–0.85, *P* = 0.001). In agreement with observations obtained in localized prostate cancer patients, the uc.368 rs8004379C variant was also significantly associated with PCSM in advanced prostate cancer patients after adjustment for age, PSA level at ADT initiation, Gleason score, stage, PSA nadir, time to PSA nadir, and treatment modality (HR 0.48, 95% CI 0.32–0.70, *P* < 0.001; Table 3 and Fig. 1D).

### Functional analyses of the rs8004379

The specific functions of UCRs are currently unknown, but a majority of these UCRs can be transcribed. Recent studies suggest that UCR expression profiles are altered during human tumorigenesis. Since secondary structure can influence RNA stability, we performed an analysis of RNA secondary structure prediction using RNA fold. The results indicate rs8004379 has a marked effect on uc.368 RNA structure, with a slight reduction (1.00 kcal/mol) in the free energy of the C allele compared to the A allele (Supplementary Fig. S1).

Furthermore, rs8004379 is also in the intron region of *NPAS3*. In order to investigate the putative function of the rs8004379 locus, we explored the Encyclopedia of DNA Elements (ENCODE) data for this region with the designated HaploReg tool for examining regulatory elements and protein binding sites. The data indicated rs8004379 and linked SNPs are situated at a locus containing enhancer histone marks, DNase hypersensitivity peaks, and possible motifs altering transcription factor binding in several cell lines (Supplementary Fig. S2A,S2B). We then investigated whether rs8004379 could affect *NPAS3* expression by using genetic variation and gene expression data from HapMap populations. Positive Spearman correlation coefficients indicated the protective C allele in rs8004379 is correlated with increased *NPAS3* expression, especially in the East Asian Japanese in Tokyo, Japan (JPT) population (*P* = 0.016, Supplementary Fig. S2C).

### Correlation of *NPAS3* expression with prostate cancer progression

**Figure 2:**
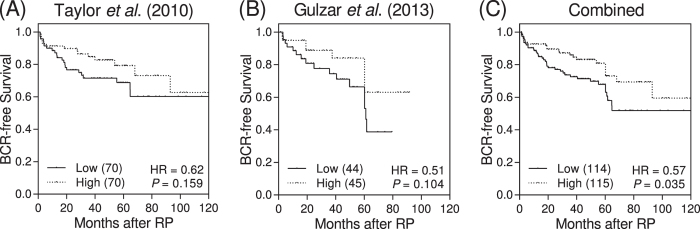
The prognostic value of *NPAS3* mRNA expression in prostate cancer. Expression of *NPAS3* mRNA is compared with survival in datasets from (**A**) Taylor *et al.* (**B**) Gulzar *et al.* and (**C**) in combined analysis. Patients were divided into high and low groups according to the median expression value of *NPAS3*. Numbers in parentheses indicate the number of patients.

We hypothesized that *NPAS3* might have an important role in prostate cancer progression. When patients were grouped by *NPAS3* expression above and below the median value, there was a trend toward correlation of increased BCR-free survival with higher *NPAS3* expression in two independent prostate cancer microarray datasets (Fig. 2A,B)^6^,^7^. In a combined analysis, increased *NPAS3* expression significantly associated with reduced recurrence after RP (*P* = 0.035, Fig. 2C).

## Discussion

In this multi-stage genetic association study, we systematically evaluated the effects of SNPs within UCRs on prostate cancer progression and mortality. We found that rs8004379 was not only significantly associated with BCR in two independent cohorts of localized prostate cancer patients, but also associated with PCSM in a cohort with advanced prostate cancer. Moreover, rs8004379 tends to have an effect on nearby *NPAS3* gene expression, with higher *NPAS3* expression in tumors correlated with better clinical outcomes, further strengthening our findings of this SNP-phenotype association.

UCRs have been found to be frequently located in genomic regions involved in cancer, and may have important functions, such as serving as enhancers, regulating splicing, regulating epigenetic modifications, or serving as transcriptional coactivators^3^,^8^,^9^,^10^,^11^. Most importantly, the majority of UCRs can be transcribed, and numerous transcribed UCRs are differentially expressed between prostate tumor and normal tissues, between tumors with high and low Gleason score, and between tumor with metastasis and those without^4^. Downregulation of some transcribed UCRs is tightly linked to the presence of CpG island promoter hypermethylation of the genes^12^. It has been found that the expression of several UCRs was recovered upon 5-AzaC, a DNA demethylating agent, treatment in prostate cancer cell lines^4^, indicating that these UCRs were epigenetically silenced in prostate cancer. Furthermore, many transcribed UCRs have significant antisense complementarity with microRNAs, which might lead to UCR-microRNA interactions and mutually regulate their expression in both directions. Calin and collaborators demonstrated a negative correlation between the expression of some UCRs and microRNAs^3^. In particular, they confirmed that uc.160 and uc.346A showed antisense complementarity with miR-155 and the expression levels of both UCRs were significantly reduced after the overexpression of miR-155 in leukemia cells. By contrast, several lines of research have also identified UCRs as a post-transcriptional modulator of microRNA function. Overexpression of uc.283A impairs the proper RNA processing machinery of pri-miR-195 and reduces the production of mature miR-195, which in turn leads to the derepression of many miR-195 targets related to cell proliferation^13^. However, there is no putative CpG island or microRNA interaction region identified around the rs8004379, according to the *in silico* analysis using MethPrimer^14^ and RegRNA^15^. Because the biological significance of UCRs remains largely unknown, and may not be uniform, it is difficult to identify the molecular function of disease-associated SNPs within UCRs. Previous studies have shown that functional noncoding regions in the human genome had conserved RNA secondary structures^16^, and certain diseases could be caused by variant-induced structural changes^17^. Our results found that rs8004379 had significant effects on a predicted uc.368 RNA secondary structure (Supplementary Fig. S1), which might alter the accessibility of the miRNAs and might have causal effects on prostate cancer progression.

The SNP rs8004379 is also located within an intron of the *NPAS3* gene, encoding a member of the neuronal PAS transcription factor gene family, which has diverse roles including neurobehavior and tumor development^18^. Functional annotations indicated that rs8004379 and linked SNPs coincide with a region of open chromatin, probably corresponding to an enhancer region for *NPAS3*, and multiple possible regulatory motifs (Supplementary Fig. S2A,S2B). The expression quantitative trait locus analysis suggested an association of the rs8004379 C allele with increased *NPAS3* expression in the East Asian JPT population (Supplementary Fig. S2C). Furthermore, gene expression survival analyses showed that higher levels of *NPAS3* correlated with improved outcomes for prostate cancer patients (Fig. 2). A recent study supported the hypothesis that *NPAS3* acts as a putative tumor suppressor during astrocytoma progression^19^. Together, these data indicate that the rs8004379 C allele might be associated with increased expression of *NPAS3* and decreased risk of recurrence and mortality in prostate cancer patients after treatment.

A major challenge in the prostate cancer care is to refine the stratification of high-risk patients and improve clinical outcomes. Our results suggest that a UCR SNP, rs8004379, confers an independent risk of prostate cancer progression compared with commonly used clinical factors (Tables 2 and 3), emphasizing the importance of UCRs on prostate cancer. The prognostic models including rs8004379 fitted significantly better than that with clinical factors only (likelihood ratio chi-square 53.6, df 1, *P* < 0.001 for localized prostate cancer; chi-square 19.4, df 1, *P* < 0.001 for advanced prostate cancer). Although this biomarker is germline, we observed altered *NPAS3* gene expression in an Asian population (Supplementary Fig. S2C). In addition, decreased expression of *NPAS3* correlated significantly with poorer BCR-free survival in independent datasets (Fig. 2). In this study, we identified that *NPAS3* might be not only a promising prognostic indicator, but also a potential therapeutic target. If validated in independent populations, our work may possibly help classify patients at higher risk of disease progression who should probably be offered early intensified therapy after initial treatment in future clinical trials. However, additional studies are required to gain better understanding of the impact of uc.368 and *NPAS3* on prostate cancer outcomes.

To our knowledge, this is the first association study using three independent cohorts to assess the influence of SNPs within UCRs on prostate cancer progression and mortality. It should be noted that the homogeneous Taiwanese population in this study may limit the generalization of these findings to other ethnic groups. However, the results are supported by the association of rs8004379 with prostate cancer progression from both localized and advanced patients, as well as the consistent *in silico* functional findings. This molecular marker might lead to a better patient stratification, and optimize therapeutic interventions in patients with a high risk of recurrence who are more likely to benefit from treatment. Further fine mapping will help to identify the causative variants responsible for the observed associations, and functional studies will also be necessary to elucidate the underlying biological mechanisms.

## Methods

### Patient recruitment and data collection

This study included 458 localized prostate cancer patients who underwent RP as their initial therapy, and 504 advanced prostate cancer patients who received primary ADT, as described previously^20^,^21^,^22^,^23^,^24^,^25^. The cohort of localized prostate cancer patients consisted of participants from two independent datasets. The discovery dataset was composed of 246 patients from the National Taiwan University Hospital, located in northern Taiwan, and the replication dataset was composed of 212 patients from the Kaohsiung Medical University Hospital, E-Da Hospital, and Kaohsiung Veterans General Hospital, all located in southern Taiwan. Demographic, clinical, and follow-up data were obtained from medical records. BCR was defined as two consecutive PSA test values of at least 0.2 ng/mL after RP^26^,^27^. The advanced prostate cancer cohort was composed of 504 patients from all four medical centers previously mentioned. PCSM was defined as the interval from initiation of ADT to death from prostate cancer. This study was approved by the Institutional Review Boards of National Taiwan University Hospital, Kaohsiung Medical University Hospital, E-Da Hospital, and Kaohsiung Veterans General Hospital. Written informed consent was obtained from each patient, and the study was carried out in accordance with approved guidelines.

### SNP selection and genotyping

We first screened common SNPs (MAF > 0.05) within UCRs by comparing 481 described UCRs^1^ with HapMap East Asian populations [Han Chinese in Beijing, China (CHB), and JPT]^28^, and identified 16 SNPs in UCRs. The SNP rs2056117 was excluded due to a strong linkage disequilibrium with rs2056116 (*r*^2^ = 0.96). Genomic DNA was extracted from peripheral blood using the QIAamp DNA Blood Mini Kit (Qiagen, Valencia, CA, USA) and stored at −80 °C until the time of study. Genotyping was performed at the National Center for Genome Medicine, Taiwan, using the Agena Bioscience iPLEX matrix-assisted laser desorption/ionization time-of-flight mass-spectrometry technology, as described previously^23^. The average genotype call rate for these SNPs was 98.1%, and the concordance rate was 100% among 10 blind duplicate quality control samples. Any SNP that significantly deviated from the Hardy-Weinberg equilibrium (*P* < 0.05), or fell below a genotyping call rate of 90%, was removed (*N* = 1, rs2303946). Thus, 14 SNPs were selected for further statistical analysis.

### Statistical analysis

Patient clinicopathologic characteristics were summarized as the number and percentage of patients in that category or median clinical values for test results with associated interquartile range. The association of clinicopathologic characteristics with time to BCR and PCSM was assessed using a log-rank test or Cox regression analysis. Multivariate Cox proportional hazard regression analysis was used to assess the effect of each SNP on clinical outcomes while adjusting for clinicopathologic variables, as previously described^27^,^29^,^30^,^31^,^32^,^33^,^34^,^35^. In the patient cohort with localized prostate cancer, explanatory variables included the known prognostic factors of age, PSA value at diagnosis, pathologic Gleason score, and tumor stage. In the advanced prostate cancer patient cohort, explanatory variables included the known prognostic factors of age, clinical stage, Gleason score, PSA at ADT initiation, PSA nadir, time to PSA nadir, and treatment modality. We compared three genetic models of inheritance to determine the significance of each SNP: dominant (common homozygotes versus variant allele carrying genotypes), recessive (common allele carrying genotypes versus variant homozygotes), and additive (*P* for trend). Only dominant and additive models were considered if variant homozygotes were observed in <0.05 of the study population. Heterogeneity between cohorts was evaluated by Cochran’s *χ*^2^-based *Q* statistical test. If the results of the *Q* test were significant, a random-effects model was used to accommodate the diversity, otherwise the combined HR was estimated using a fixed-effects model. SPSS software, version 22.0.0 (IBM, Armonk, NY, USA), was used for statistical analyses. A two-sided *P* value of <0.05 was considered statistically significant.

### Bioinformatics analysis

We used several bioinformatics tools to assess whether rs8004379 and its linked genetic variants were associated with a putative function affecting patient outcomes. HaploReg v3^36^ and the ENCODE^37^ data were used to identify regulatory features of the regions adjoining the SNPs. The association of rs8004379 with *NPAS3* expression was evaluated using mRNA data from lymphoblastoid cell lines derived from 90 Utah residents with northern and western European ancestry (CEU), 45 CHB, 45 JPT, and 90 Yoruba in Ibadan, Nigeria (YRI), HapMap individuals^38^. The publicly available datasets^6^,^7^ were used to analyze *NPAS3* expression and prostate cancer outcomes.

## Additional Information

**How to cite this article**: Bao, B.-Y. *et al.* Genetic variants in ultraconserved regions associate with prostate cancer recurrence and survival. *Sci. Rep.* **6**, 22124; doi: 10.1038/srep22124 (2016).

## Supplementary Material

Supplementary Information

## Figures and Tables

**Table 1:** Clinical characteristics of study cohorts.

Characteristic	Discovery	Replication	Combined	*P*^ a^
Localized prostate cancer cohort				
Patients, n	246	212	458	
Age at diagnosis				0.149
Median, y (IQR)	65 (61–69)	68 (62–71)	66 (61–70)	
PSA at diagnosis				<0.001
Median, ng/mL (IQR)	10.1 (6.7–15.8)	12.6 (7.6–19.8)	11.1 (7.1–17.5)	
≤20	192 (81.7)	155 (76.0)	347 (79.0)	
>20	43 (18.3)	49 (24.0)	92 (21.0)	
Pathologic Gleason score, n (%)				<0.001
≤7	217 (89.7)	175 (82.9)	392 (86.5)	
>7	25 (10.3)	36 (17.1)	61 (13.5)	
Pathologic stage, n (%)				<0.001
T1/T2	173 (72.4)	130 (61.3)	303 (67.2)	
T3/T4/N1	66 (27.6)	82 (38.7)	148 (32.8)	
BCR	75 (30.5)	109 (51.4)	184 (40.2)	
Median follow-up time^b^, mo (95% CI)	50 (45–55)	60 (56–64)	54 (50–58)	
Advanced prostate cancer cohort				
Characteristic		*P*^ c^		
Patients, n	504			
Age at diagnosis		0.904		
Median, y (IQR)	73 (66–79)			
≤72	250 (49.6)			
>72	254 (50.4)			
PSA at ADT initiation		<0.001		
Median, ng/mL (IQR)	33.8 (9.3–133.3)			
Biopsy Gleason score at diagnosis, n (%)		<0.001		
<7	139 (28.3)			
7	173 (35.2)			
>7	180 (36.6)			
Clinical stage at diagnosis, n (%)		<0.001		
M0	308 (61.4)			
M1	194 (38.6)			
PSA nadir		<0.001		
Median, ng/mL (IQR)	0.14 (0.01–1.06)			
<0.2	275 (54.8)			
≥0.2	227 (45.2)			
Time to PSA nadir		<0.001		
Median, mo (IQR)	10 (5–20)			
Treatment modality		0.002		
ADT as primary treatment	254 (50.5)			
ADT for post RP PSA failure	73 (14.5)			
ADT for post RT PSA failure	12 (2.4)			
Neoadjuvant/adjuvant ADT with RT	122 (24.3)			
Others	42 (8.3)			
PCSM	94 (18.7)			
Median follow-up time^b^, mo (95% CI)	60 (57–63)			

Abbreviations: IQR, interquartile range; PSA, prostate-specific antigen; BCR, biochemical recurrence; CI, confidence interval; ADT, androgen deprivation therapy; RP, radical prostatectomy; RT, radiation therapy; PCSM, prostate cancer-specific mortality.

^a^*P* value was calculated by the log-rank test or Cox regression for BCR in combined 458 localized prostate cancer patients.

^b^Median follow-up time and 95% CIs were estimated with the reverse Kaplan-Meier method.

^c^*P* value was calculated by the log-rank test or Cox regression for PCSM in advanced prostate cancer patients.

**Table 2:** Association of rs8004379 with BCR in localized prostate cancer patients treated with RP.

SNP Genotype	Discovery			Replication				Combined		
	No BCR, n (%)	BCR, n (%)	HR (95% CI)^a^	*P*^ a^	No BCR, n (%)	BCR, n (%)	HR (95% CI)^a^	*P*^ a^	HR (95% CI)^a^	*P*^ a^
rs8004379										
AA	74 (43.3)	45 (60.0)	1.00		42 (41.6)	54 (50.9)	1.00		1.00	
AC	77 (45.0)	23 (30.7)	0.52 (0.31–0.90)	0.018	44 (43.6)	46 (43.4)	0.78 (0.52–1.18)	0.241	0.67 (0.49–0.92)	0.01
CC	20 (11.7)	7 (9.3)	0.47 (0.20–1.14)	0.096	15 (14.9)	6 (5.7)	0.40 (0.17–0.94)	0.035	0.43 (0.24–0.79)	0.007
AC/CC vs AA			0.51 (0.31–0.84)	0.008			0.70 (0.47–1.05)	0.083	0.62 (0.45–0.84)	0.002
CC vs AA/AC			0.62 (0.26–1.47)	0.278			0.45 (0.20–1.04)	0.061	0.52 (0.29–0.95)	0.03
Trend			0.61 (0.41–0.91)	0.015			0.70 (0.51–0.96)	0.027	0.66 (0.52–0.85)	0.001

Abbreviations: BCR, biochemical recurrence; RP, radical prostatectomy; SNP, single nucleotide polymorphism; HR, hazard ratio; CI, confidence interval; PSA, prostate-specific antigen.

*P* < 0.05 is in boldface.

^a^Adjusted by age, PSA at diagnosis, pathologic Gleason score, and pathologic stage.

**Table 3:** Association of rs8004379 with PCSM in advanced prostate cancer patients treated with ADT.

SNP Genotype	No PCSM, n (%)	PCSM, n (%)	5-year survival rate, %	HR (95% CI)^a^	*P *a
rs8004379					
AA	173 (42.7)	46 (48.9)	76.3	1.00	
AC	190 (46.9)	42 (44.7)	80.9	0.43 (0.27–0.68)	<0.001
CC	42 (10.4)	6 (6.4)	88.3	0.29 (0.11–0.74)	0.010
AC/CC vs AA				0.40 (0.26–0.63)	<0.001
CC vs AA/AC				0.46 (0.19–1.15)	0.096
Trend				0.48 (0.32–0.70)	<0.001

Abbreviations: PCSM, prostate cancer-specific mortality; ADT, androgen deprivation therapy; HR, hazard ratio; CI, confidence interval; PSA, prostate-specific antigen.

*P* < 0.05 is in boldface.

^a^Adjusted by age, clinical stage, Gleason score, PSA at ADT initiation, PSA nadir, time to PSA nadir, and treatment modality.
