# Supplementary material for: Genetic variants in ultraconserved regions associate with prostate cancer recurrence and survival
Source: Sci Rep. 2016 Feb 23;6:22124. doi: 10.1038/srep22124 (PMC4763269; doi:10.1038/srep22124)
Supplement: Supplementary Information [file srep22124-s1.pdf]

Genetic variants in ultraconserved regions associate with prostate cancer recurrence and survival

Bo-Ying Bao[1,2,3], Victor C. Lin[4,5], Chia-Cheng Yu[6,7,8], Hsin-Ling Yin[9,10], Ta-Yuan Chang[11], Te-Ling Lu[1], Hong-Zin Lee[1], Jiunn-Bey Pao[12], Chao-Yuan Huang[13,*], Shu-Pin Huang[14,15,16,*]

[1]Department of Pharmacy, China Medical University, Taichung, Taiwan, [2]Sex Hormone Research Center, China Medical University Hospital, Taichung, Taiwan, [3]Department of Nursing, Asia University, Taichung, Taiwan, [4]Department of Urology, E-Da Hospital, Kaohsiung, Taiwan, [5]School of Medicine for International Students, I-Shou University, Kaohsiung, Taiwan, [6]Division of Urology, Department of Surgery, Kaohsiung Veterans General Hospital, Kaohsiung, Taiwan, [7]Department of Urology, School of Medicine, National Yang-Ming University, Taipei, Taiwan, [8]Department of Pharmacy, Tajen University, Pingtung, Taiwan, [9]Department of Pathology, Kaohsiung Medical University Hospital, Kaohsiung, Taiwan, [10]Department of Pathology, Faculty of Medicine, College of Medicine, Kaohsiung Medical University, Kaohsiung, Taiwan, [11]Department of Occupational Safety and Health, China Medical University, Taichung, Taiwan, [12]Department of Pharmacy, Linsen Chinese Medicine Branch, Taipei City Hospital, Taipei, Taiwan, [13]Department of Urology, National Taiwan University Hospital, College of Medicine, National Taiwan University,

Taipei, Taiwan, [14]Department of Urology, Kaohsiung Medical University Hospital, Kaohsiung, Taiwan, [15]Department of Urology, Faculty of Medicine, College of Medicine, Kaohsiung Medical University, Kaohsiung, Taiwan, [16]Graduate Institute of Medicine, College of Medicine, Kaohsiung Medical University, Kaohsiung, Taiwan

[*]Correspondence and requests for materials should be addressed to C.Y.H. (cyhuang0909@ntu.edu.tw) or S.P.H. (shpihu@yahoo.com.tw)

Supplementary Table S1 | Genotyped SNPs and the *P* values of their association with BCR after RP

UCR	SNP ID	Chromosome	Position	BCR		
				Additive	Dominant	Recessive
uc.51	rs17049105	2	57972610	0.184	0.133	-
uc.53	rs1861100	2	59133512	0.421	0.37	0.912
uc.82	rs13020355	2	156727173	0.505	0.346	0.842
uc.83	rs11896224	2	156991899	0.693	0.564	0.88
uc.133	rs2682406	3	158026488	0.747	0.728	0.889
uc.140	rs2056116	4	13009986	0.678	0.642	0.888
uc.252	rs1538101	9	16710869	0.913	0.803	-
uc.268	rs12981	9	125607010	0.578	0.655	0.073
uc.269	rs1752168	9	126537951	0.064	0.089	-
uc.295	rs7092999	10	102375257	0.719	0.924	0.556
uc.302	rs11190870	10	102979206	0.087	0.272	0.097
uc.353	rs9572903	13	72771713	0.364	0.355	0.685
uc.368	rs8004379	14	34068700	0.055	**0.020**	0.705
uc.374	rs7143938	14	37716057	0.208	0.141	0.624

Abbreviations: SNP, single nucleotide polymorphism; BCR, biochemical recurrence; RP, radical prostatectomy; UCR, ultraconserved region.

P values for log-rank test.

P < 0.05 is in boldface.

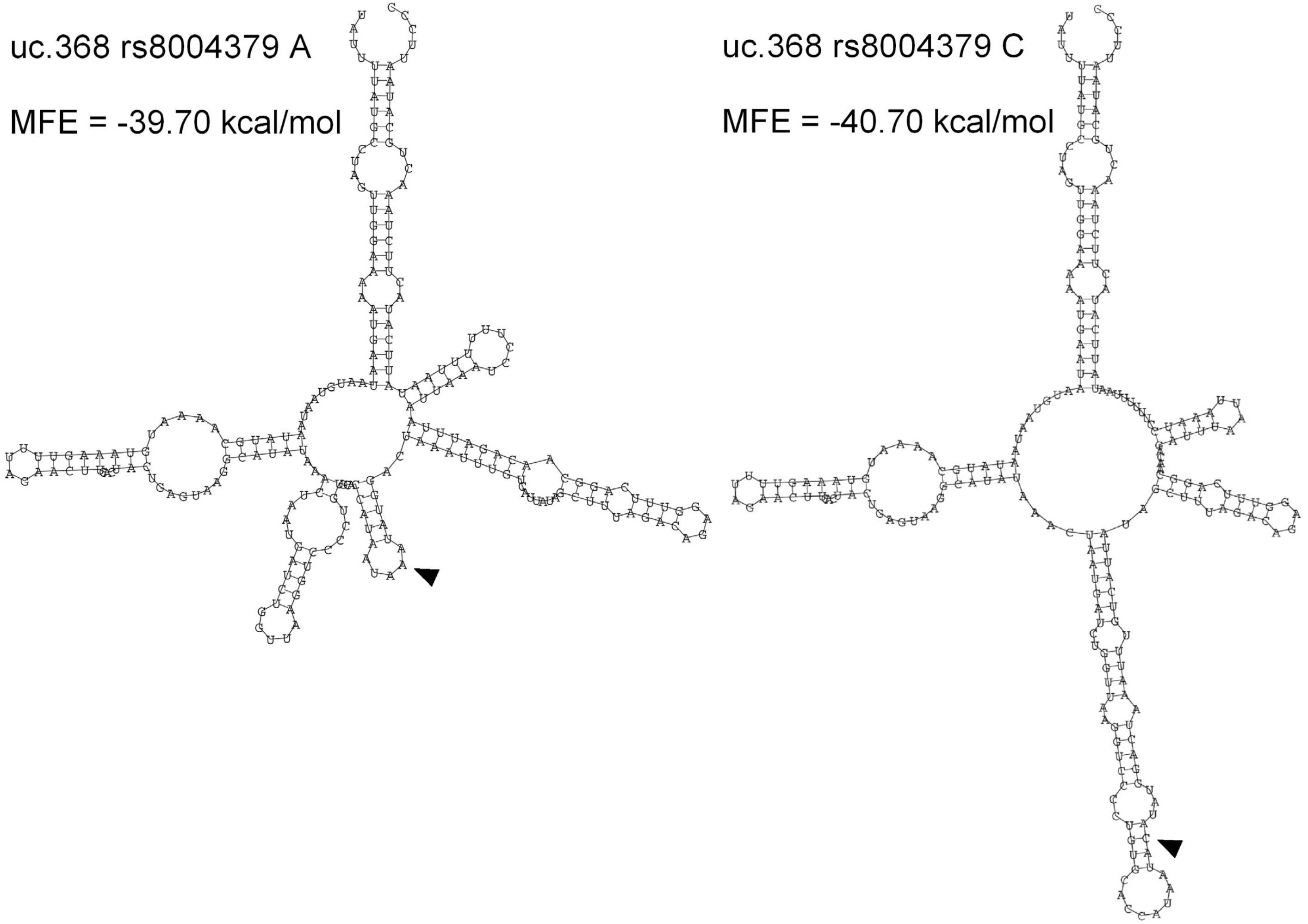

Supplementary Figure S1 | rs8004379 A>C generates a different RNA secondary structure for uc.368. The minimum free energy (MFE) RNA secondary structures were predicted by RNAfold. Arrows indicate the position of the polymorphism within the secondary structures.

(A)

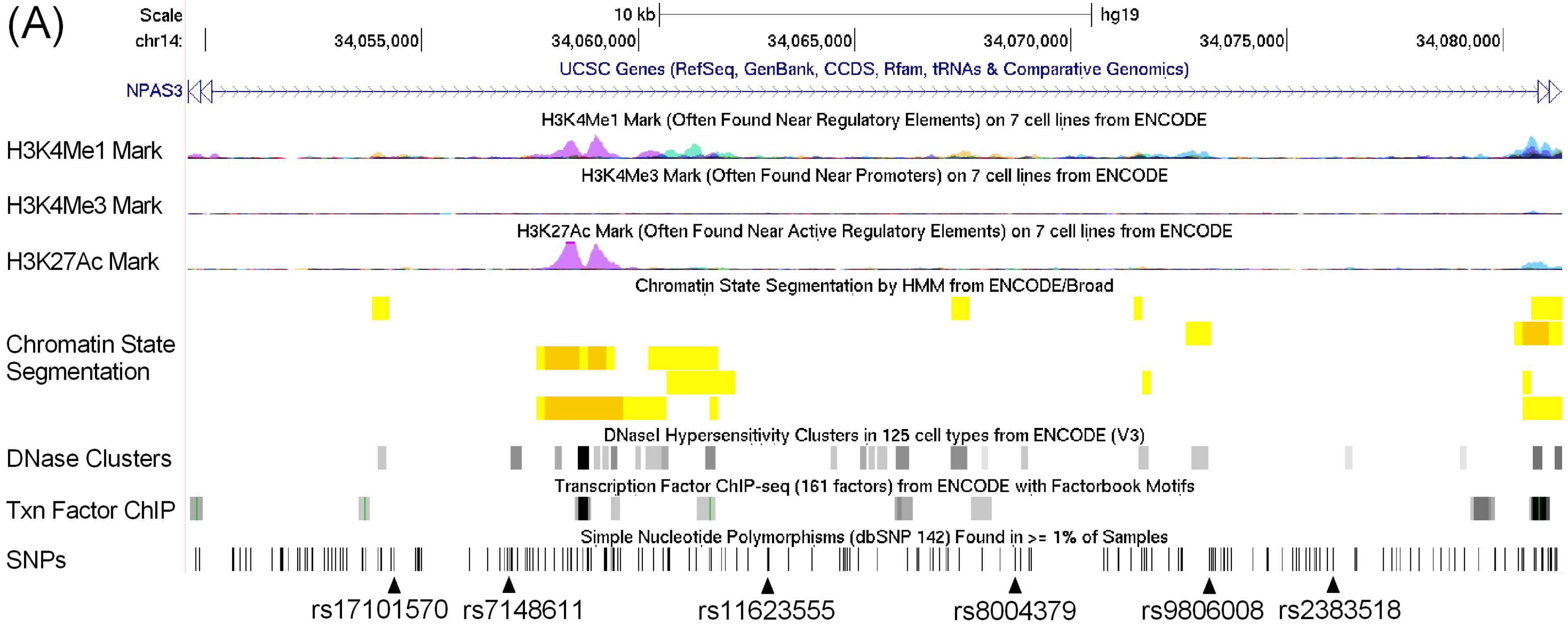

(B)

SNP ID	Allele	LD (r^2)	Variant type	Enhancer histone marks	DNase	Motifs changed
rs17101570	T/C	0.88	*NPAS3* intronic	ESDR, IPSC		Hic1
rs7148611	A/C	0.92	*NPAS3* intronic		SKIN	Hlx1, Hoxc9, Pou2f2
rs11623555	T/G	0.96	*NPAS3* intronic			Tgif1
rs8004379	A/C	1	*NPAS3* intronic	ESC, PANC		Foxa, Nkx6-2, TATA
rs9806008	G/A	0.83	*NPAS3* intronic	MUS		AP-1, Hoxb4, Nkx2, Nobox, OTX, Prrx2, Sox
rs2383518	T/C	0.89	*NPAS3* intronic	BLD	BLD, BLD, SKIN	Arid5b, FAC1

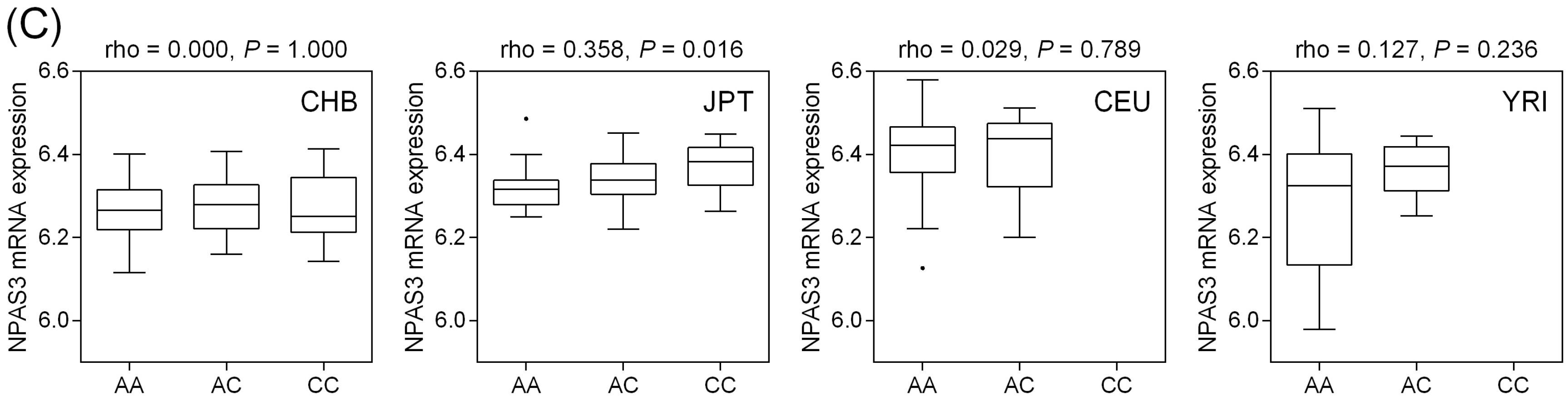

Supplementary Figure S2 | Summary of the functional analyses for the linkage disequilibrium (LD) block containing *NPAS3* rs8004379. (A) Expanded view of the ENCODE data for the LD block containing the *NPAS3* rs8004379. The H3K4Me1, H3K4Me3, and H3K27Ac tracks show the genome-wide levels of enrichment of the mono-methylation of lysine 4, tri-methylation of lysine 4, and acetylation of lysine 27 of the H3 histone protein, as determined by the ChIP-seq assays. These levels are thought to be associated with promoter and enhancer regions. Chromatin State Segmentation track displays chromatin state segmentations by integrating ChIP-seq data using a Hidden Markov Model for H1 embryonic stem cells, HUVEC umbilical vein endothelial cells, HMEC mammary epithelial cells, HSMM, skeletal muscle myoblasts, and NHEK epidermal keratinocytes. The chromatin state regions predicted for promoters and enhancers are highlighted. DNase clusters track shows DNase hypersensitivity areas. Tnx Factor track shows regions of transcription factor binding of DNA, as assayed by ChIP-seq experiments. (B) Regulatory annotation of variants within the LD block containing *NPAS3* rs8004379. In the LD block with the lead SNP rs8004379, HaploReg database showed evidence of enhancer elements coinciding with the variants in many different cell types. In addition, Foxa, Nkx6-2, and TATA motifs are predicted to be affected. (C) Expression quantitative trait locus analysis of *NPAS3* expression relative to the genotype for rs8004379 in HapMap populations. *NPAS3* expression was associated with rs8004379 genotype in Japanese population. rho, Spearman's rank correlation coefficient.
